# The topology of pen-2, a γ-secretase subunit, revisited: evidence for a reentrant loop and a single pass transmembrane domain

**DOI:** 10.1186/s13024-015-0037-4

**Published:** 2015-08-22

**Authors:** Xulun Zhang, Chunjiang J. Yu, Sangram S. Sisodia

**Affiliations:** Department of Neurobiology, The University of Chicago, 947 E. 58th St. MC0928, Chicago, Il 60637 USA; Department of Neurobiology, The University of Chicago, 1128 S. Eastside Ave, Oak Park, Chicago, Il 60304 USA

**Keywords:** Pen-2, Topology, γ-secretase

## Abstract

**Background:**

The γ-secretase complex, composed of transmembrane proteins termed presenilin (PS), anterior pharynx defective (APH), nicastrin (NCT), and presenilin enhancer-2 (Pen-2) catalyzes intramembranous hydrolysis of a variety of Type I membrane protein substrates. In order to understand aspects of subunit assembly, interactions, dynamics and catalysis, it is essential to clarify the membrane topology of each polypeptide. Hydophathicity plots predict that the 101 amino acid Pen-2 molecule has two hydrophobic domains (HP1 and HP2) that may serve as transmembrane spanning domains. Earlier reports indicated that transiently overexpressed Pen-2 uses these two hydrophobic domains as transmembrane helices that generates a “U-shaped” hairpin topology with both amino- (N-) and carboxyl-(C-) termini facing the lumen. In this report, we have reexamined the topology of endogenous Pen-2 and Pen-2 chimeras that are stably expressed in mammalian cells, and have assessed the function of these molecules in rescuing γ-secretase activity in *Pen-2*-deficient fibroblasts.

**Results:**

We confirm that the Pen-2 C-terminus is lumenal, but the N-terminus of Pen-2 is exposed to the cytoplasm, thus indicating that HP1 does not traverse the lipid bilayer as a transmembrane domain. Domain swapping studies reveal the importance of specific regions within the first hydrophobic domain of Pen-2 that are critical for generating the topology that is a prerequisite for mediating PS1 endoproteolysis and γ-secretase activity. Finally, we report that the first fourteen amino acids of the Pen-2 HP1 are required for γ-secretase activity.

**Conclusions:**

We propose that the first hydrophobic domain of Pen-2 forms a structure similar to a reentrant loop while the second hydrophobic domain spans the lipid bilayer.

## Background

The γ-secretase complex catalyzes intramembranous proteolysis of several type I membrane proteins, including the amyloid-beta precursor protein (APP) and Notch (for review, see Ref.[1]) and is composed of presenilins (PS1 and PS2), anterior pharynx defective (APH-1), and presenilin enhancer-2 (Pen-2) and the Type I membrane protein, nicastrin (NCT) [2–4]. During the assembly of active γ-secretase, Aph-1 and NCT form a stable subcomplex that binds to, and stabilizes the PS holoprotein/zymogen [5, 6] via binding to the PS1 C-terminus [7]. Pen-2 then binds to PS1 in the trimeric APH-1-NCT-PS1 complex, and promotes the proteolytic conversion of the PS holoprotein into ~37-40 kDa N- and ~17-20 kDa C-terminal fragments that are the preponderant PS1 polypeptides that accumulate *in vivo* [5, 6, 8–13]. The steady-state levels of each subunit of the γ-secretase complex is coordinately regulated and dependent on the expression of other components of the complex [14].

The topology of PS1 [15] and Aph-1 [16] have been determined. Hydopathicity plots of Pen-2 indicate the presence of two hydrophobic regions (HP1 and HP2) that could serve as potential transmembrane domains (TMD) [2]. We reported that the proximal two-thirds of the Pen-2 HP1 is functionally required for endoproteolysis of PS1 holoproteins and the generation of PS1 fragments, while the C-terminal hydrophilic domain of Pen-2 is critical for stabilizing the PS1 NTF- and CTF, but is not required for PS1 endoproteolysis [17]. In this regard, it has been reported that Pen-2 assumes a “U-shaped” hairpin topology wherein the HP regions serve as TMDs and both the N- and C-terminal domains are exposed to the lumen [18, 19].These conclusions were based on analysis of Pen-2 chimeras with engineered N-glycosylation sites that were transiently expressed in mammalian cells. Three complementary approaches were employed: examination of *N*-glycosylation site usage to demonstrate lumenal exposure, immunofluorescence detection by selective membrane permeabilization agents (Streptolysin O (SLO) or Triton X-100), and protease protection assays. Similarly, we reported that proteinase K treatment of membranes prepared from HEK293 cells transiently cotransfected with cDNA encoding PS1, NCT, APH-1 and Pen-2 resulted in the protection of ~6 kDa carboxyl- (CTF) and ~4 kDa amino-terminal (NTF) fragments derived from Pen-2, suggesting that the molecule adopts a “U-shaped” hairpin topology [17]. Unfortunately, there are several experimental caveats that would lead to misinterpretation of the outcomes reported by Crystal et al. [18] and Bergman et al. [19], the most significant of which is that the functional activity of the overexpressed Pen-2 variants in mediating PS1 endoproteolysis or γ-secretase activity were never assessed. Furthermore, the fact that steady-state accumulation of each component of the γ-secretase complex is strictly regulated by the levels of other subunits [14], transient overexpression would lead to unproductive populations of unassociated subunits that are likely to be misfolded and hence, could adopt alternate topologies, or differential membrane insertion characteristics. In this regard, we reported that while detectable levels of Pen-2 NTF and CTF were protected from proteinase K digestion of membranes from HEK293 cells that transiently coexpressed PS1, NCT, APH-1 and Pen-2, the levels of these fragments were extremely low. Instead, the bulk of Pen-2 remained as full-length molecules even up to 25 ug/ml of enzyme. Moreover, solubilization of this membrane preparation by the addition of Triton-X100, prior to the addition of 25 ug/ml proteinase K, completely eliminated the full-length Pen-2 signal [17], a finding that would suggest that the vast majority of Pen-2 expressed in this overexpression setting was lumenally disposed, a scenario that we consider highly unlikely. Thus, we felt it was important to investigate the topology of endogenous Pen-2 or Pen-2 that are stably expressed in mammalian cells. In the present report, we confirm that the C-terminus of Pen-2 is lumenally disposed, but in contrast to earlier studies, we document that the N-terminus resides in the cytoplasm. Furthermore, we demonstrate that a subregion within HP1 is critical for determining the topology of Pen-2 that is required for facilitating PS1 endoproteolysis and γ-secretase activity [17].

## Results and discussion

### Pen-2 does not exhibit a “U-shaped” hairpin topology

**Fig. 1:**
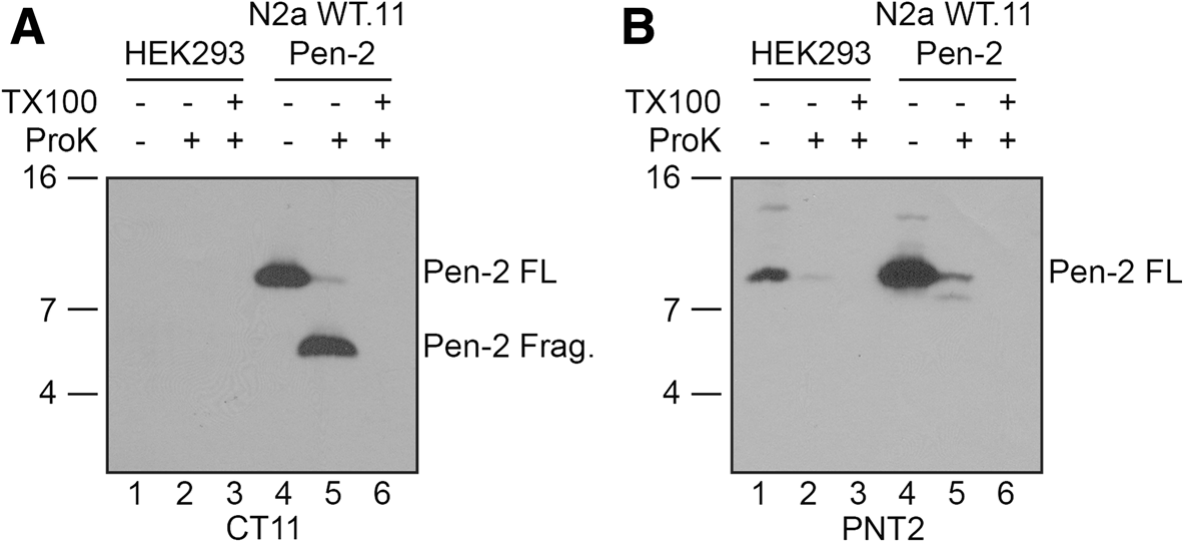
The amino-terminus of Pen-2 is in the cytoplasm and the carboxyl-terminus is in the lumen. 10 μg of protein from P15 fractions from HEK 293 cells (Lanes 1–3) or stable N2aWT.11 cells expressing CT11-tagged Pen-2 (Lanes 4–6) were incubated with Proteinase K in the absence or presence of Triton X-100. Resulting reactions were fractionated in 16.5 % Tris/Tricine SDS-PAGE and analyzed by Western blotting with CT11 (**a**. Lanes 1–6) or PNT-2 (**b**. Lanes 1–6) antibodies. Molecular mass markers are shown on the left in kilodaltons

Standard hydropathicity plots reveal that Pen-2 harbors two hydrophobic regions (HP1 and HP2) separated by a 21 amino acid spacer. When expressed in transiently transfected cells, it has been reported that Pen-2 adopts a “U-shaped” hairpin topology with both N- and C-termini facing the membrane lumen [17–19]. In view of the possibility that transiently overexpressed membrane proteins may adopt multiple protein conformations and topologies, we reexamined Pen-2 topology in HEK293 cells that express Pen-2 endogenously or in N2aWT.11 cells [8] that stably express wild-type human Pen-2 harboring a C-terminal CT-11 epitope tag [17]. We prepared 15,000 x *g* (P15) membrane fractions from these cells, and treated the material with proteinase K in the absence or presence of 1 % Triton X-100. The reaction products were analyzed by SDS-PAGE and Western blot analysis with the CT11 antibody or the PNT-2 antibody generated against the first 26 residues of PEN-2 [11]. Proteinase K treatment of the membrane fractions from N2aWT.11/Pen-2 cells lead to an ~5 kDa CT11-positive C-terminal fragment (CTF) that was protected from digestion (Figure 1a, lane 5), while co-incubation of a parallel mixture with Proteinase K together with Triton X-100, a detergent that disrupts all membranes, eliminated this fragment (Fig. 1a, lane 6). These results indicate that in N2aWT.11 cells expressing CT11-tagged Pen-2, the CT11 epitope resides in the lumen, a result consistent with earlier findings [18]. Unfortunately, an antibody specific for the native Pen-2 C-terminus is not available, and hence, the disposition of Pen-2 expressed endogenously in HEK293 could not be determined. We then probed Western blots of protease-treated membranes with the PNT-2 antibody (Fig. 1b). In protease-treated HEK293 membranes (Fig. 1b, lane 2) or N2aWT.11/Pen-2 membranes (Fig. 1b, lane 5), we observe that the full-length Pen-2 signal is eliminated, but surprisingly, we failed to detect the predicted 4-kDa amino-terminal fragment (NTF) that would have been protected if this region faced the lumen. This result suggests that the amino-terminus of Pen-2 is sensitive to the proteinase K, arguing that the epitope(s) detected by PNT-2 are located in the cytoplasm. In summary, these latter studies do not support the “U-shaped” topology model that was proposed earlier, and argue instead for a model in which the Pen-2 N-terminus is cytosolic and the C-terminus is lumenal.

The results presented above would suggest that Pen-2 must adopt a topology consistent with an odd (either one or three) number of TMDs. If HP1 was utilized as the *sole* TMD, then we would have expected an ~10 kDa C-terminal, CT11-positive protected fragment after protease treatment of N2aWT.11/Pen-2 membranes. However, our demonstration that an ~5 kDa protected CTF is observed in protease-treated N2aWT.11/Pen-2 membranes can only be consistent with HP2 serving as the sole TMD.

**Fig. 2:**
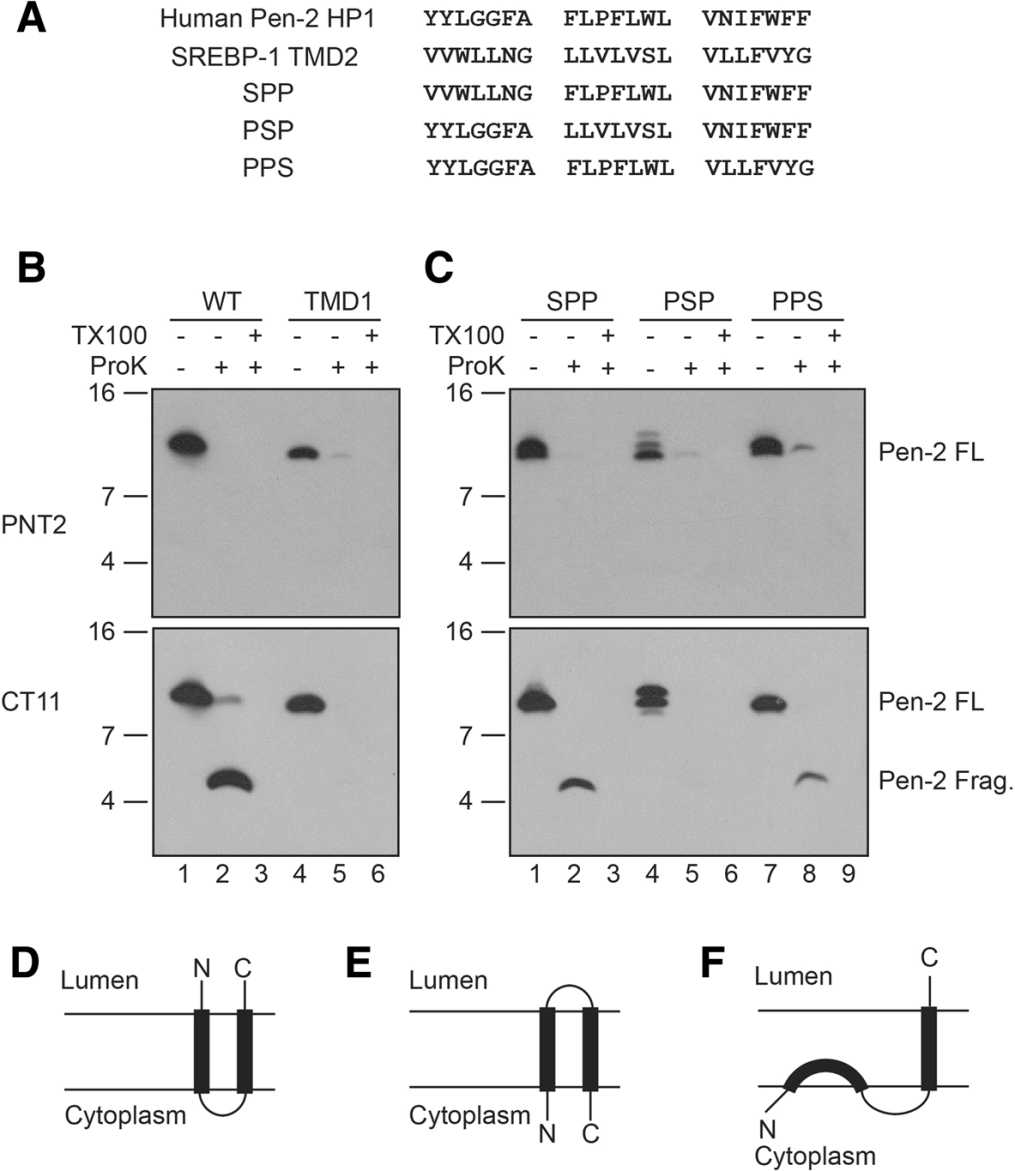
The first hydrophobic region of Pen-2 is not a TMD. **a**. Sequence of Pen-2 HP1, SREBP TMD2 and Pen-2-SREBP chimeras. **b**. Cell membranes from stable cell lines expressing CT11 tagged Pen-2 (lanes 1–3) or Pen-2 TMD1 chimera (lanes 4–6) were treated with proteinase K in the absence or presence of Triton X-100 and resulting products were subject to immunoblotting with PNT2 (upper panel) or CT11 (lower panel) antibodies. **c**. Cell membranes from Pen-2 chimeras with substitutions of segments from the SREBP TMD2 were treated with proteinase K in the absence or presence of Triton X-100 and resulting products were subject to immunoblotting with PNT2 (upper panel) or CT11 (lower panel) antibodies. Molecular mass markers are shown on the left in kilodaltons. **d**. Pen-2 model proposed in Refs 17, 18 and 19. E. Model for Pen-2 TMD1. F. Revised Pen-2 model

In earlier studies, we examined the function of a CT11 epitope-tagged Pen-2 chimera, TMD2-SREBPPen-2, in which the Pen-2 HP2 was replaced with the first TMD of the sterol regulatory binding element binding protein-1 (SREBP). SREBP exhibits a “bell-shaped” hairpin topology, and hence we chose to replace Pen-2 HP2 with the TMD1 of SREBP in order to maintain the native Type I membrane orientation. We demonstrated that this chimeric molecule retains Pen-2 function in mediating endoproteolysis of PS1 and γ-secretase activity [17] and hence, the amino acid sequence of HP2 *perse* was not a functional determinant. Because the first HP region of SREBP is a *bona fide* TMD, it follows that HP2 of Pen-2 is likely to be a TMD, as well, a conclusion supported by the protection of a CT11-positive ~5 kDa CTF after protease digestion of membranes from N2aWT.11/Pen-2 cells. Taken together with the results shown in Fig. 1, which revealed that the N-terminus is sensitive to protease digestion, we felt it was implausible that the Pen-2 HP1 region could also serve as a TMD. Nevertheless, we tested the possibility that the Pen-2 HP1 could serve as a TMD, as was previously reported [17–19] by generating a stable cell line expressing “TMD1”, a SREBP/Pen-2 chimera in which HP1 of Pen-2 was replaced with TMD2 of SREBP that has a Type II membrane orientation (Fig. 2a). We had previously established that this fusion protein is not functional with respect to promoting PS1 endoproteolysis or γ-secretase activity [17]. Moreover, this fusion protein does not coimmunoprecipitate with PS1 [17], a result which indicated that the Pen-2 HP1 mediates binding to PS1. To determine the topology of TMD1, we prepared membranes from an N2aWT.11 cells that stably expresses this chimera, then treated this preparation with proteinase K and performed Western blot assays with PNT-2 or CT11 antibodies. We neither detected protected NTF derived from wild-type Pen-2, as expected, nor from the TMD1 chimera, indicating that the N-termini of these molecules are cytoplasmic (Fig. 2b, upper panel, lanes 2 and 5, respectively). In contrast, while proteinase K generated a protected ~5 kDa CT11-positive CTF derived from wild-type Pen-2 (Fig. 2b, lower panel, lane 2), we did not observe a protected CTF derived from the TMD1 chimera (Fig. 2b, lower panel, lane 5). We interpret this result to suggest that replacement of the Pen-2 HP1 with the authentic TMD2 of SREBP now generates a chimera with 2 TMDs wherein both the N- and C-termini face the cytoplasm and that the 21 amino acid spacer separating the TMDs that would otherwise reside in the cytosol would now be recruited into the lumen. As a consequence, in the TMD1 chimera, the Pen-2 HP2 would be forced to span the membrane in a Type II orientation that is opposite from that predicted for native Pen-2, thus resulting in a “bell-shaped” hairpin topology, similar to the membrane topology of SREBP and depicted in Fig. 2, Model E. This drastic change of topology also explains the loss of function for the TMD1 chimera [17]. While this model is satisfying, the membrane disposition of the Pen-2 HP1 remained uncertain.

### Molecular features of the pen-2 HP1

Since the TMD2-SREBPPen-2 chimera was functional, thus arguing that the sequence of the Pen-2 HP2 was dispensable for function [17], we reasoned that the sequence of HP1 must confer function to Pen-2. Indeed, we had demonstrated that the Pen-2 HP1 is necessary for interacting with PS1 [17]. In order to examine the topology of the Pen-2 HP1, we designed a strategy to rescue the activity of the functionally inactive CT11 epitope-tagged TMD1 chimera (14). To this end, we replaced single or multiple seven amino acid segments of the SREBP TMD2 with analogous segments of the Pen-2 HP1[17] (Fig. 2a). We showed that the only chimera to fully rescue PS1 endoproteolysis and γ-secretase activity was PPS (P = Pen-2; S = SREBP), in which the first fourteen amino acids of SREBP TMD2 was replaced with the first fourteen amino acids of Pen-2 HP1[17].

We sought to determine the topology of the functional PPS chimera or two functionally inactive chimeras, termed SPP and PSP [17] as a surrogate assay for functionality. We prepared membranes from N2a cells that stably express CT11 epitope-tagged PPS, SPP and PSP chimeras, then treated the membranes with proteinase K and subject resulting products to Western blot analysis. Using PNT-2 antibody, we show that the N-terminal epitope(s) in all three chimeras are sensitive to proteolysis, as a protected NTF was undetectable (Fig. 2c, top panel, lanes 2, 5, 8). Thus, the N-termini of these molecules must face the cytoplasm. Interestingly, like wild-type Pen-2 (Fig. 2b, lane 2), an ~5 kDa CT11-positive CTF was also protected in membranes expressing the SPP and PPS chimeras (Fig. 2c, bottom panel, lanes 2 and 8, respectively), indicating that the C-termini of these two proteins face the lumen. However, we did not observe a protected CTF derived from PSP (Fig. 2c, bottom panel, lane 5), suggesting that this region is protease-sensitive, and hence, must face the cytoplasm. We suggest that in the PSP chimera, the hybrid HP1 behaves as a true TMD that spans the membrane, thus resulting in a topology similar to the TMD1chimera or SREBP, itself, that exhibit a “bell-shaped” hairpin topology (Fig. 2, Model E). This hairpin topology model of PSP may explain the inability of this chimera to rescue the loss of function observed for the TMD1chimera [17].

**Fig. 3:**
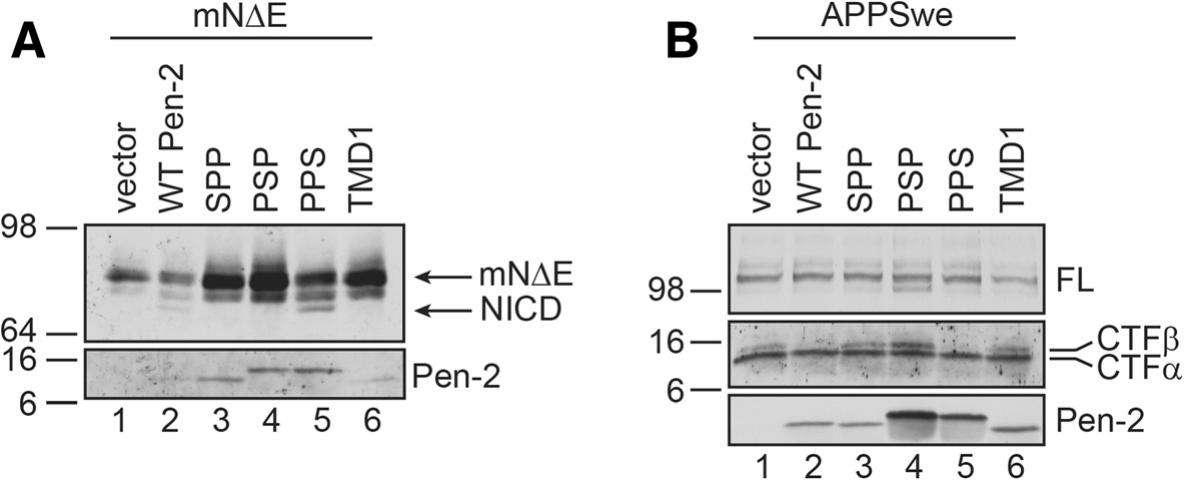
Rescue of γ-secretase Function in *Pen-2*-deficient fibroblasts by Pen-2 and the PPS chimera. **a**. mNΔE Processing in transiently cotransfected cells. **b**. APPswe processing in transiently cotransfected cells

Our earlier demonstration that the PPS chimera was functional with respect to PS1 endoproteolysis and γ-secretase activity [17], while the SPP and PSP were not, left unresolved the importance of the domain(s) within the first fourteen amino acids on the Pen-2 HP1 that are responsible for function. We sought to establish the importance of this region by asking whether we could rescue γ-secretase activity in cells that lack expression of Pen-2. For these studies, we obtained *Pen-2*-deficient fibroblasts that exhibit no detectable γ-secretase activity and in which the trimeric APH-1-NCT-PS1 intermediate complex is readily observed by Blue Native (BN)-PAGE analysis [20]. We transiently cotransfected *Pen-2*-deficient fibroblasts with cDNA encoding a well-established γ-secretase substrate, termed mouse Notch 1 lacking the ECD segment (mNΔE) [21] and cDNAs encoding wild-type Pen-2, SPP, PSP, PPS or TMD1 (Fig. 3a). We observe that NICD is not produced in *Pen-2* deficient fibroblasts transfected with the parental vector of the *Pen-2* constructs (Fig. 3a, lane 1), but that expression of wild-type Pen-2 and PPS rescued NICD production (Fig. 3a, lanes 2, 5 respectively), while the SPP, PSP and TMD1 chimeras did not (Fig. 3a, lanes 3, 4 and 6, respectively). To establish that these latter results could be extended to another γ-secretase substrate, we transiently cotransfected *Pen-2*-deficient fibroblasts with cDNA encoding the FAD-linked APP “Swedish” variant (APPswe) together with cDNAs encoding wild-type Pen-2, SPP, PSP, PPS or TMD1 (Fig. 3b). As expected, high levels of APP-CTFs were observed in cells transfected with cDNA encoding APPswe and parental (empty) vector (Fig. 3b, lane 1); notably, the α-CTF derivatives are generated primarily from endogenous APP and to a lesser degree from APPswe, while the β-CTF is derived primarily from APPswe that must be present only in the small fraction of fibroblasts that were transiently transfected. We observed a significant reduction of β-CTF levels by expression of wild-type Pen-2 and the PPS chimera (Fig. 3b, lanes 2, 5 respectively), while expression of the SPP, PSP and TMD1 chimeras failed to do so (Fig. 3b, lanes 3, 4 and 6, respectively). The reduction in levels of β-CTF by expression of wild-type Pen-2 or the PPS chimera support the view that γ-secretase activity is elevated in cells that express these polypeptides. In summary, these functional assays in transiently transfected *Pen-2*-deficient fibroblasts fully support our earlier functional studies in HEK293 cells transiently transfected with PS1, APH-1, NCT and Pen-2 or the Pen-2-SREBP chimeras [17]. More importantly, it is quite clear that separation of the first seven amino acids and the middle seven amino acids of HP1 of Pen-2 by an intervening segment is functionally intolerant, but that these regions need to be in contiguity (as in PPS) in order to restore function.

## Conclusions

Defining the topology, molecular interactions and dynamics of the putative transmembrane helices of PS, Aph-1 and Pen-2 are critical for understanding the assembly and mechanism(s) underlying the catalytic activity of γ-secretase. While the topology of PS1 and APH-1 have been established, the reported topology of Pen-2 existing as a “U-shaped” hairpin was equivocal because the exogenous proteins being examined were transiently overexpressed [17–19], conditions that may lead to the production of misfolded polypeptides with multiple topologies, mixed membrane insertion properties and subunit interactions. We sought to eliminate these potential artefacts by examining the topology of Pen-2 expressed endogenously, or in cell lines that stably express Pen-2 or a panel of Pen-2 chimeras and we now offer several important insights.

First, we show that Pen-2 expressed endogenously or in stable cell lines *does not* adopt a “U-shaped” hairpin, as was described earlier [17–19] .Using proteinase K protection assays, we confirm that the C-terminus faces the lumen, but that the N-terminus is sensitive to proteolysis, thus indicating that this region faces the cytoplasm. Notably, we attempted to validate this conclusion using detergents that selectively permeabilize plasma and/or intracellular membranes combined with immunofluorescence approaches using the N-terminal-specific PNT-2 antibody, but failed to detect the epitope (data not shown), thus arguing that this domain is buried. This finding is fully consistent with studies from our own laboratory [17] and others [19, 22] showing that antibodies specific for the amino-terminal region of Pen-2 fail to coimmunoprecipitate PS1-NTF and components of the γ-secretase complex from membranes solubilized with mild detergents including DDM or CHAPSO. These findings received further support from chemical accessibility labeling studies which revealed that a large and charged sulfhydryl-specific reagent failed to modify experimentally introduced cysteine residues in this region [20].

Second, we demonstrate that a stably expressed Pen-2 chimera, TMD1, in which the Pen-2 HP1 is replaced by TMD2 of SREBP, exhibits a “U-shaped” hairpin structure which is most likely the reason that this chimera is functionally inactive in mediating PS1 endoproteolysis and γ-secretase activity. Third, we examined the topology of a panel of stably expressed Pen-2-SREBP chimeras wherein short segments of the SREBP TMD2 were replaced with analogous peptides from the Pen-2 HP1. In view of the fact that the PPS chimera was functional, while the PSP chimera was not, we speculate that the peptide sequence in the middle third of the Pen-2 HP1, FLPFLWL, must play a critical role in insuring that Pen-2 adopts the correct topology. Indeed, comparison of this region of human Pen-2 and the analogous region from Pen-2 in phylogenetically divergent species, including *M. musculus*, *D. rario* (zebrafish), *D. melanogaster* and *C. elegans* reveals that the “FLP F/L V/L/ W” peptide sequence is highly conserved, and it is likely that the presence of the proline residue (Pro27) in the center of HP1 would act to “kink” the α-helix [23]. Interestingly, Bammens et al. [20] have shown that the Pen-2 P27C mutant accumulated to very low levels when stably expressed in *Pen-2*-deficient cells that also correlated with low levels of γ-secretase complex levels. Since proline residues have a restricted backbone that can significantly affect protein structure, we would argue that this residue has an impact both on the stability of Pen-2 and the γ-secretase complex. While plausible, our conclusion that P27 and the central region of the Pen-2 HP1 is required for function is tempered by the fact that SPP, a chimera with topological similarities to wild-type Pen-2 (i.e., N-termini facing the cytosol and C-termini facing the lumen), is functionally inactive in mediating PS1 endoproteolysis and γ-secretase activity [17]. The only resolution to this paradox is that the middle third of Pen-2 HP1 is necessary for determining the overall topology of HP1, but that both the sequence and conformation of the first fourteen amino acids of the Pen-2 HP1 are essential for binding to PS1, presumably through interactions with the “NF” motif within TM4 of PS1, that we and others determined to be necessary and sufficient for Pen-2 binding [17, 24, 25].

In summary, we have demonstrated that Pen-2 harbors a single membrane spanning TMD corresponding to HP2, with the C-terminus exposed to the lumen. Moreover, we also demonstrate that the N-terminus of Pen-2 faces the cytoplasm and by inference, HP1 does not span the membrane bilayer. Our model is illustrated in Fig. 2f, and is supported by an elegant report from the laboratory of Yigong Shi and colleagues [26]wherein the cryo-EM structure of human γ-secretase at 4.32-Å resolution has recently been determined. This level of resolution allows specific assignment of all TMs in the complex and subunit packing. This structural analysis reveals that Pen-2 harbors not two, but three TMDs, the first two of which traverse the membrane only half-way from the intracellular side [26]. Thus, the Pen-2 HP1 is partially buried, but does not traverse, the lipid bilayer of mammalian membranes in a manner similar to “reentrant” loops, defined as a structural motif that goes only halfway through the membrane and then turns back to the side from which it originates and that are a signature of transporters/ion channels [27]. Moreover, and as was predicted from earlier mutagenesis and biochemical studies [17, 24], the cyo-EM studies reveal that N-terminal region of the Pen-2 HP1 associates with TM4 of PS1.

Future studies using mutagenesis and crosslinking approaches, in combination with *in silico* modeling and high resolution X-ray crystallography will be required to define the molecular basis for Pen-2-mediated autocatalytic endoproteolysis of PS1 [9] and “activation” of γ-secretase activity. Clarification of these outstanding issues may lead to the development of novel strategies to modulate of γ-secretase activity.

## Methods

### Cell culture and transfection

Human embryonic kidney (HEK) 293 cells were cultured in 5 % CO_2_ at 37 °C in Dulbecco's modified Eagle's medium (DMEM) containing 100 units/ml penicillin and 100 μg/ml streptomycin sulfate, supplemented with 10 % (v/v) fetal bovine serum (FBS). Mouse neuroblastoma N2a cells stably expressing human presenilin 1 (N2aWT.11 cells) and Pen-2 or various TMD1-SREP Pen-2 chimeras [17] were maintained in 50 % DMEM and 50 % of Opti-MEM contining 200 ug/ml of hygromycin, 100 units/ml penicillin and 100 μg/ml streptomycin sulfate, supplemented with 5 % FBS. Immortalized *Pen-2*-deficient fibroblasts [20] were generously provided by Dr. Bart DeStrooper (University of Leuven, Belgium). *Pen-2*-deficient cells were transiently transfected using Lipofectamine 2000 (Life Technologies).

### Constructs

The cDNAs encoding CT11-tagged Pen-2 and its variants were described previously [17].

### Cell fractionation

Monolayers of HEK293 cells or stable N2a cells were grown to subconfluence, scraped into 3 ml phosphate-buffered saline with 5 mM EDTA, and centrifuged at 1000 *g* for 5 min at 4 °C. The cell pellet from each dish was resuspended in 0.4 ml sucrose-containing buffer H (10 mM Hepes-KOH at pH 7.4, 10 mM KCl, 1.5 mM MgCl_2_, 5 mM sodium EDTA, 5 mM sodium EGTA, 250 mM sucrose), passed through a 22-gauge needle 20 times, and centrifuged at 1000 *g* for 5 min at 4 °C. Each supernatant was centrifuged at 1.5 × 10^4^ *g* for 10 min at 4 °C, and the resulting pellet was designated as the P15 membrane fraction.

### Protease protection assay

P15 membrane fractions from HEK293 cells or N2a stable lines expressing PS1, and either wild-type Pen-2 or the TMD1-SREBP Pen-2 chimeras were treated with proteinase K in the presence or absence of 1 % Triton X-100 [18]. After incubation at room temperature for 90 min, the reactions were terminated by the addition of phenylmethylsulfonyl fluoride (PMSF) and subject to centrifugation at 100,000 x *g* for 1 h. The pellet fraction was washed once with Buffer H, resuspended in Laemmli SDS sample buffer and fractionated by 16.5 % Tris/Tricine SDS/PAGE.

### Western blot

Cell lysates and protein fragments were fractionated in Tris/glycine or 16.5 % Tris/Tricine SDS-PAGE, and transferred to a nitrocellulose membrane (Schleicher and Schuell) prior to incubation with appropriate primary and secondary antisera. Immunoreactive proteins were visualized using an enhanced chemiluminescence detection system (ECL; PerkinElmer Life Sciences).

### Antibodies

The following antibodies were used for this study and have been described previously [17] . The PNT-2 antibody was raised against a peptide corresponding to the NH_2_-terminal 26 amino acids of Pen-2 (a generous gift from Dr. Gopal Thinakaran). The Rb mAb PEN2 is from Abcam (ab154830). The CT11 antibody specifically recognizes the last 7 amino acids (RFLEERP) of APLP1.

## Abbreviations

APH-1: Anterior pharynx defective
APP: β-Amyloid precursor protein
CTF: Carboxyl-terminal fragment
NCT: Nicastrin
NTF: Amino-terminal fragment
Pen-2: Presenilin enhancer
PMSF: Phenylmethylsulfonyl fluoride
PS: Presenilin
SREBP: Sterol regulatory element binding protein-1
TMD: Transmembrane domain
WT: Wild type
CHAPSO: 3-[(3-Cholamidopropyl)dimethylammonio]-2-hydroxy-1-propanesulfonic acid
HEK293 cells: Human embryonic kidney 293
Tricine: *N*-[2-Hydroxy-1,1 bis(hydroxymethyl)ethyl]glycine
DDM: n-Dodecyl-β-D-maltopyranoside
